# Research collaboration data platform ensuring general data protection

**DOI:** 10.1038/s41598-024-61912-8

**Published:** 2024-05-24

**Authors:** Monica Toma, Caroline Bönisch, Benjamin Löhnhardt, Michael Kelm, Hanibal Bohnenberger, Sven Winkelmann, Philipp Ströbel, Tibor Kesztyüs

**Affiliations:** 1 Siemens Healthineers AG, Erlangen, Germany; 2 Medical Data Integration Center, Department of Medical Informatics, University Medical Center Göttingen, Göttingen, Germany; 3 Faculty of Electrical Engineering and Computer Science, University of Applied Sciences Stralsund, Stralsund, Germany; 4 Department of Medical Informatics, University Medical Center Göttingen, Göttingen, Germany; 5 Nuremberg Institute of Technology, Nuremberg, Germany; 6 Institute of Pathology, University Medical Center Göttingen, Göttingen, Germany

**Keywords:** Medical research, Translational research

## Abstract

Translational data is of paramount importance for medical research and clinical innovation. It has the potential to benefit individuals and organizations, however, the protection of personal data must be guaranteed. Collecting diverse omics data and electronic health records (EHR), re-using the minimized data, as well as providing a reliable data transfer between different institutions are mandatory steps for the development of the promising field of big data and artificial intelligence in medical research. This is made possible within the proposed data platform in this research project. The established data platform enables the collaboration between public and commercial organizations by data transfer from various clinical systems into a cloud for supporting multi-site research while ensuring compliant data governance.

## Introduction

Translational data is of paramount importance for medical research and clinical innovation. The combination of different omics data (e.g., genomics, radiomics, proteomics) and clinical health data with big data analytics and artificial intelligence (AI) has the potential to transform the healthcare to a proactive P4 medicine that is predictive, preventive, personalized, and participatory^1^. Based on this potential, medical research builds on data, which should be easily findable, accessible, interoperable, and re-usable (FAIR) for (secondary) use^2^. Unfortunately, clinical health data is usually stored in so called data or information silos. These silos are prone to restrict access and reuse of the data by holding disparate data sets^3–5^. However, to enable AI, an extraordinarily large amount of data is needed to train or model the neural network^6^. For this purpose, data must be collected and curated appropriately besides being stored (professionally) so that it is reliable for (further) exploitation^7^. In medical areas like pathology or radiology, where diagnostics rely on medical imaging managed by the Digital Imaging and Communications in Medicine (DICOM) standard, a large amount of data can be collected during examination and treatment, while machine learning is already well established^8–10^.

The Medical Informatics Initiative (MII)^11^, funded by the German Ministry of Education and Research (BMBF) is a joined collaboration project, connecting various German university hospitals, research institutions, and businesses while overcoming enclosed clinical health data silos and exchange medical information. To create interoperable research frameworks, different consortia have been formed, within the MII. Every consortium established a Medical Data Integration Center (MeDIC) at German university hospitals to bridge and merge data from different clinical source systems. This pooling of data is mostly done within a (research) data platform. A data platform is a set of technologies that enables the acquisition, storage, curation, and governance of data while ensuring security for its users and applications. With the integration of multi omics and electronic health records (EHR), important information can enrich the information in a health data platform^12^.

Additionally, most of the clinical health data within university hospitals contain personal data and are therefore subject to specific privacy protection laws and regulations. Within the European Union (EU) the data protection directive from 1995 was replaced by the General Data Protection Regulation (GDPR) (https://gdpr.eu/tag/gdpr/.) in 2016. The GDPR applies to organizations everywhere if they deal with data related to people in the EU. In contrast to the previous legislation, the country-specific protection laws were harmonized within the GDPR. The Regulation now contains detailed requirements for commercial and public organizations when collecting, storing, and managing personal data.

The GDPR sets the scene for a lawful, fair, and transparent usage of data in the Article 5, defining the principles related to processing of personal data. The data should only be collected for specified purposes (purpose limitation) and should be minimized accordingly (data minimization). The personal data should rely on accuracy, integrity, and confidentiality, while storage limitations should be carefully considered. The GDPR also specifies the principle of accountability, according to which the controller shall be responsible for processing of personal data in compliance with the principles of the GDPR and able to demonstrate this compliance as in the Article 5.2 of the GDPR. Especially when processing is based on the data subject’s consent, the controller should be able to demonstrate this consent as defined in the Article 7.1 of the GDPR. Furthermore, the purpose-based consent should be, not only informed and specific, but unambiguous as well, so that the data subject’s wishes are reflected in the processing of personal data.

Each of the principles under GDPR Article 5 apply to all data processing, including research purposes. Scientific research is seen, as an important area of public interest, hence derogations from the general rules are provided in Article 89 of the GDPR. Within a valid legal basis and subject to the principle of proportionality and appropriate safeguards, secondary use of research data is possible: “those measures may include pseudonymization provided that those purposes can be fulfilled in that manner. Where those purposes can be fulfilled by further processing which does not permit or no longer permits the identification of data subjects, those purposes shall be fulfilled in that manner”^13^. Nevertheless, secondary use of data in research projects remain a gray area in this field^14–16^. Especially if considering to use the data by third parties, like commercial partners.

According to GDPR, data minimization refers to the regulation of personal data being “adequate, relevant, and limited to what is necessary in relation to the purposes for which they are processed”^17^. This means, that data beyond this scope should not be included in the data collection and analysis process. Because of the data minimization requirements, it is essential for data platforms to retain the scope for every data set, making it possible to refer to the scope at every step of the data processing. Especially the area of data minimization is essential for the use of data platforms, because of the potential threat of violation of user’s privacy by combining data independently from a dedicated scope or use case^18^.

**The challenge.** Based on the aforementioned GDPR regulation requirements, that need to be taken into account, processing of data from the data platform for research purposes necessitates transferring data from clinical data storage into the cloud considering data privacy issues and legal constraints in a collaborative research environment. A crucial aspect of this work is providing a reliable data transfer between different institutions, while ensuring compliance with data privacy regulations, e.g., GDPR. Despite the data being minimized locally by the university medical site, the purpose limitation is ensured.

## Methods

### Literature review

To contextualize the work presented, the authors executed a comprehensive literature review. Literature databases, such as PubMed and Embase were searched for publications on the topic of the article with a restriction to English and German language in a period from January 2016 to January 2024. The search strategy was created by combining database specific index terms (e.g., Emtree - Embase Subject Headings) and free terms relevant to the aim of the study with Boolean operators as shown in Table 1. In result, 103 hits from both databases PubMed and Embase could be obtained by the literature search. Proceeding from these hits, 67 matches were considered after the title and abstract screening. Continuing from this step, all full texts of these hits were retrieved and examined. After reviewing the full texts, further 18 results could be excluded. The remaining matches form the foundation of the work presented and are addressed inter alia in the Section Related Work.

**Table 1:** Search strategy on 08.01.2024 in Embase.

**Number**	**Search Step**	**Results**
1	Big data/ or information processing/ or information retrieval/ or electronic medical record system/ or cloud computing/ or clinical infrastructure.mp. data lake.mp. or research platform.mp	326,473
2	Medical research/ or clinical research/ or translational research/ or data analysis/ or data science/ or information dissemination/ or data sharing.mp. or artificial intelligence/ or medical informatics research.mp.	628,436
3	Computer security/ or data protection/ or identifiable information/ or confidentiality/ or (general data protection regulation or “GDPR”).mp. or privacy/ or legal aspect/ or law/ or data minimization.mp. or data minimisation.mp.	312,373
4	Pseudonymisation.mp. or FAIR principles/ or “FAIR data”.mp. or cloud.mp. or secondary data analysis/ or “secondary use”.mp.	29,821
5	1 and 2 and 3 and 4	208
6	Limit 5 to (human and (english or german) and yr=“2016 -Current” and (article or article in press or chapter or conference paper or “review”))	96

### Related work

The work in this research project is closely related to (research) data platform approaches which exchange data (on) through cloud services between different stakeholders including both commercial and public organizations.

Froehlicher et al.^19^ proposes an encrypted, federated learning approach, to overcome the hurdle of processing privacy-protected data within a centralized storage. In differentiation, the rationale of this present article includes centralized data for research purposes, while considering GDPR compliant strategies to make data interoperable and accessible and neglecting technical workarounds to bypass the GDPR requirements.

A technical solution similar to the results presented in this article is shown by Bahmani et al.^20^. They present a platform for minimized data which is transferred from an app e.g., wearables to a cloud service. However, it must be noted that the approach of Bahmani et al. does not include a legal framework in relation to data privacy and the inclusion of a data contract.

The commentary of Brody et al. introduces a “cloud-based Analysis Commons”^21^, a framework that combines genotype and phenotype data from whole-genome sequencing, which are provided via multiple studies, by including data-sharing mechanism. Although this commentary provides a likely similar approach to bridge the gap between data collection within multiple studies and data transfer and interoperability to an analytical platform it does not touch on the implementation of the framework in compliance with data privacy principles as required by the GDPR. The exchange of data is secured via a “consortium agreement rather than through the typical series of bilateral agreements”^21^ to share data across institutions.

The research data portal for health (“Forschungsdatenportal für Gesundheit”) developed within the MII^22^ was made available in September 2022.The portal is currently running within a pilot phase and allows researchers to apply centrally for health data and biological samples for scientific studies.The data to be queried is based on a core data set^23^ that was developed within the MII. The proposed approach in this manuscript allows both clinical researchers associated with university hospitals as well AI researchers associated with industrial partners to work together on the same dataset at the same time. Furthermore, the data available in the described research platform is already cleared by the ethic committee of the organization uploading the data, so that a new vote is not necessary in contrary to the approach taken by the MII. Furthermore, the exchange between the university hospital and the commercial partner is provided via a data contract with specific data governance measures including rights and permissions. This data contract is registered in the cloud prior to a data transfer.

Continuing from the MII, the Network of University Medicine (NUM), established in 2020,^24,25^ contributes through its coordinated efforts and platforms to better prepare German health research and, consequently, the healthcare system as a whole for future pandemics and other crises. NUM, started as part of crisis management against COVID-19, coordinating clinical COVID-19 research across all university hospital sites, fostering collaboration among researchers for practical, patient-centric outcomes and better management of public health crises.The sub-project Radiological Cooperative Network (RACOON) is the first of its kind to bring together all university departments of a medical discipline and establish a nationwide platform for collaborative analysis of radiological image data^26–28^. This platform supports clinical and clinical-epidemiological studies as well as the training of AI models. The project utilizes technology allowing structured data capture from the outset, ensuring data quality, traceability, and long-term usability. The collected data provide valuable insights for epidemiological studies, situational assessments, and early warning mechanisms. Within RACOON the Joint Imaging Platform (JIP) established by the German Cancer Consortium (DKTK) incorporates federated data analysis technology, where the imaging data remain at the site, where it originated and the analysis algorithm are shared within the platform. JIP provides a unified infrastructure across radiology and nuclear medicine departments of 10 university hospitals in Germany. A core component is “SATORI”, a browser-based application for viewing, curating and processing medical data. SATORI supports images, videos and clinical data, with particular benefits for radiological image data. While this project is very promising and brings great potential, it is designed for radiological data and images, while the project adressed in this research manuscript is laying its focus on pathology data. Furthermore, the exchange with an industrial partner differs from the network partners listed for RACOON (university radiology centers and non-university research institutes).

Another additional infrastructure for the exchange of federated data is GAIA-X^29^. GAIA-X aims to exchange data in a trustworthy environment and give users control over their data. The GAIA-X infrastructure is based on a shared model with the components - data ecosystems and infrastructure ecosystems. Data is exchanged via a trust framework with a set of rules for participation in GAIA-X. This approach differs from the approach written in the manuscript in the form of the data contract between the partners involved and the pseudonymization of the data during exchange.

The results of the literature search led to the conclusion that there are few comparable approaches of research data platforms which exchange medical data via a cloud. However, no identical approaches could be identified. In particular, the exchange of data under consideration of a data contract in relation to a legal framework regarding GDPR could not be found amongst the research results.

### Clinical infrastructure and data minimization

To ensure the exchange of medical data while considering GDPR regulations between a MeDIC - the network used in this research project, is divided using network segmentation to handle data with a higher protection class accordingly. The clinical systems (e.g., pathology systems) are located in the so-called patient network segment (PatLAN) of the research facility and is kept separated from the research network segment (WissLAN). In regards of keeping the data stored, to a minimum, a data minimization step is performed in the staging layer between the patient network segment and the research network segment. Only data items required for further processing are transferred between the two networks. In regards of collecting the data it could have been useful and advised to use the broad patient consent (as established within the MII) in such a research project. But at the start of research project presented in 2022, it had not been introduced at the UMG at that time. The underlying patient consent is recorded manually on paper, but was afterwards be entered digitally by a study nurse and passed through into the study data pool within the UMG-MeDIC. From there it is then provided to the industrial partner, as part of the data shared. It includes consent to data release and further processing within the study mentioned in Section “Methods”. After collecting the patient consents, personal data is replaced by a pseudonymization process. Here, an independent trusted third party (TTP) takes over the task of replacing the personally identifiable data (PII) with a pseudonym (unique generated key code). This pseudonymization can only be reversed by the TTP. This TTP is established at the MeDIC. Mapping tables of personal data and assigned pseudonyms are exclusively known to the TTP and the TTP can, if medically advised and if there is a corresponding consent for re-contact, carry out a de-pseudonymization. The staff of the TTP office is released from the authority of the MeDIC executive board regarding the pseudonymization of personal data. The TTP staff is the only party that could perform the process of de-pseudonymization based on a documented medical reason.

### Cloud infrastructure

The described current status under Section “Clinical infrastructure and data minimization” with regard to the clinical infrastructure and the approach of making the data available for analysis via a cloud infrastructure made it necessary to deal with cloud services that enable sharing big data in medical research. Efficient data management is more important than ever, helping businesses and hospitals to gain analytical insights, as well as using machine learning in the medical field (e.g., to predict molecular alterations of tumors^30^). The first generation of big data management mainly consisted of data warehouses which provided storage for structured data. Over time as more unstructured data emerges and is stored within clinical infrastructures, a second generation of big data management platforms called data lakes were developed. They incorporate low-cost (cloud) storages (e.g., Amazon Simple Storage Service, Microsoft Azure Storage, Hadoop Distributed File System) while holding generic raw data.

**Figure 1:**
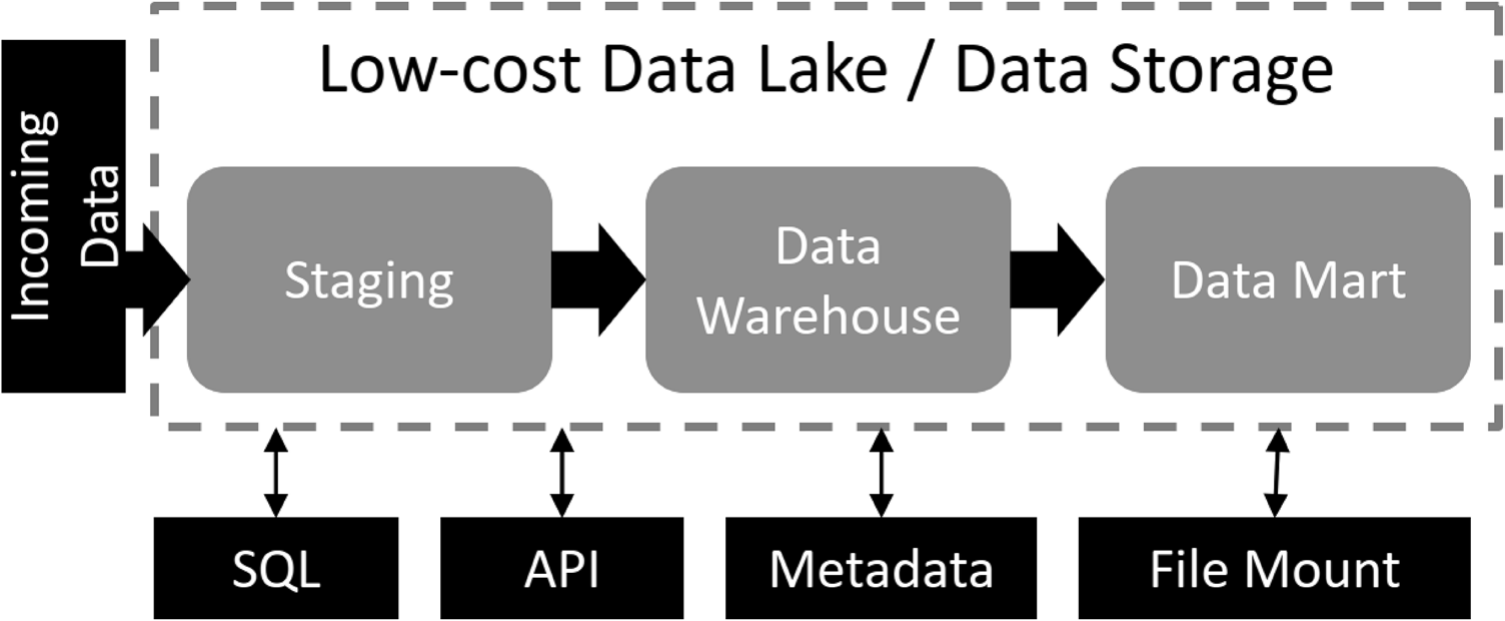
Three-layered cloud infrastructure with uniform data access. Figure based on^31^ and^32^.

Although combining data from warehouses and data lakes is highly complex for data users, Zaharia et al.^32^ propose an integrated architecture which combines a low-cost data lake (cf. Fig. 1) with direct file access and performance features of a data warehouse and database management system (DBMS) such as: atomicity, consistency, isolation, durability (ACID) transactions, data versioning, auditing, and indexing on the storage level. All these components can be combined with a three-layer clustering (cf. Fig. 1) which is usually used for data warehouses: staging area (or bronze layer) for incoming data, data warehouse (or silver layer) for curated data, and data access (or gold layer, or data mart) for end users or business applications^31,33,34^. The cloud infrastructure used, is structured accordingly, to enhance the benefits of this three-layer clustering.

### Ethic review

Ethical approval for the study was obtained from the Ethics Review Committee of the University Medical Center Göttingen (Ref No. 24/4/20, dated 30.04.2020), and all developments and experiments were performed in accordance with relevant guidelines and regulations. Furthermore, informed consent was obtained from all subjects and/or their legal guardian(s).

## Results

**Figure 2:**
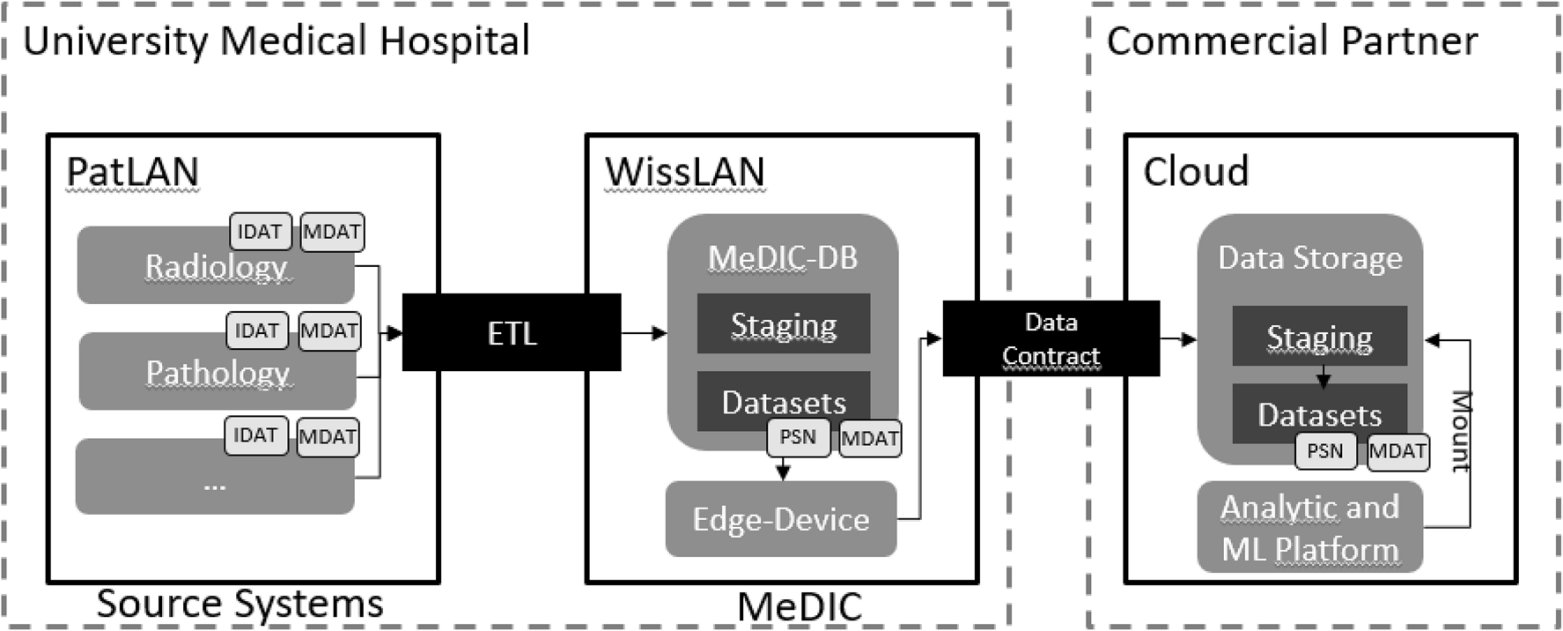
System overview to transfer data from clinical systems to the cloud providing access for commercial partners.

Establishing a data transfer from clinical data storage of a MeDIC into a cloud requires connecting different source systems. A system overview of the approach is shown in Fig.  2. Firstly, data retrieved from clinical systems (segment PatLAN as patient network segment, which is separated from the internet) is processed and saved in the MeDIC (segment MeDIC as part of the research network segment WissLAN). Secondly, the data is transferred from the MeDIC to the cloud with a software component (called edge-device) that ensures authentication and data encryption. The solution proposed in this research project is based on European Privacy Seal-certified cloud products (https://euprivacyseal.com/de/eps-en-siemens-healthcare-teamplay/  [Online 2024/01/16].) to be privacy compliant with the GDPR. The approach was validated by the development, testing, and deployment of a novel AI tool to predict molecular alterations of tumors^30^ based on the data transferred from one clinical institution.

### Clinical infrastructure

The data transfer from the PatLAN segment to the MeDIC (and vice versa) is only possible under certain conditions. The University Medical Center Göttingen (UMG) established the MeDIC as a data platform to integrate the data from the clinical source systems in patient care (e.g., pathology). Subsequently the collected data is processed and made available in different formats to different infrastructures (e.g., cloud) depending on the use case.

As described in Fig. 2 the data in the source systems contain both personal data (IDAT) and the medical data (MDAT). The MeDIC receives the data from the clinical source systems in a particular network segment within PatLAN. In this step of the operation the data is handled by an ETL (extract, transform, load) process for data minimization and transformation^35^. This means that only the minimized data is stored in the MeDIC. This step replaces the personal data (IDAT) with a pseudonym (PSN) as a prerequisite for being able to process the data in the research network. Only by means of the trusted third party (as described in Section “Clinical infrastructure and data minimization”) the pseudonyms can be resolved back to an actual person. Additionally, this information is not transferred to the research network segment (WissLAN). In case of a consent withdrawal, the medical data involved will be deleted from all storage locations at the MeDIC and commercial partner. To ensure the revocation, an automatic process is initiated. The process performs the deletion of the data within the MeDIC and triggers a deletion process to the commercial partner by sending the pseudonyms (PSN) to be deleted.

### Data contract

After the data is processed within the clinical infrastructure of the MeDIC, the data, received by the commercial partner will be stored after a so called “data contract”, which is designed as a questionnaire, that specifies data governance measures, including rights and permissions. For the provision of the data via the edge-device to the cloud infrastructure, the minimized data from the MeDIC will be used. It is submitted and registered on the cloud prior to the data transfer. The “data contract” includes a data protection impact assessment (DPIA) by design to assess the re-identification risks that may arise from the content and context of the data aggregation. A data owner affiliated with the commercial partner will be assigned to a specific data set. The data owner must therefore ensure that the data is processed in compliance with the purpose stipulated in the legal obligations.The data contract triggers the correct distribution and storage of data in the respective regional data center. Moreover, only designated parties can process the data to the extent which is necessary for the permitted purpose. Logs to all data activities are provided. The period of storage and usage is defined including the obligations to cite the origin of the data or to disclose the results generated by the data usage. Furthermore, it is ensured that when a request to delete a specific data set is received (e.g., withdrawal of consent) it is possible to track this data and remove it completely in a timely manner.

### Cloud infrastructure supporting research

**Figure 3:**
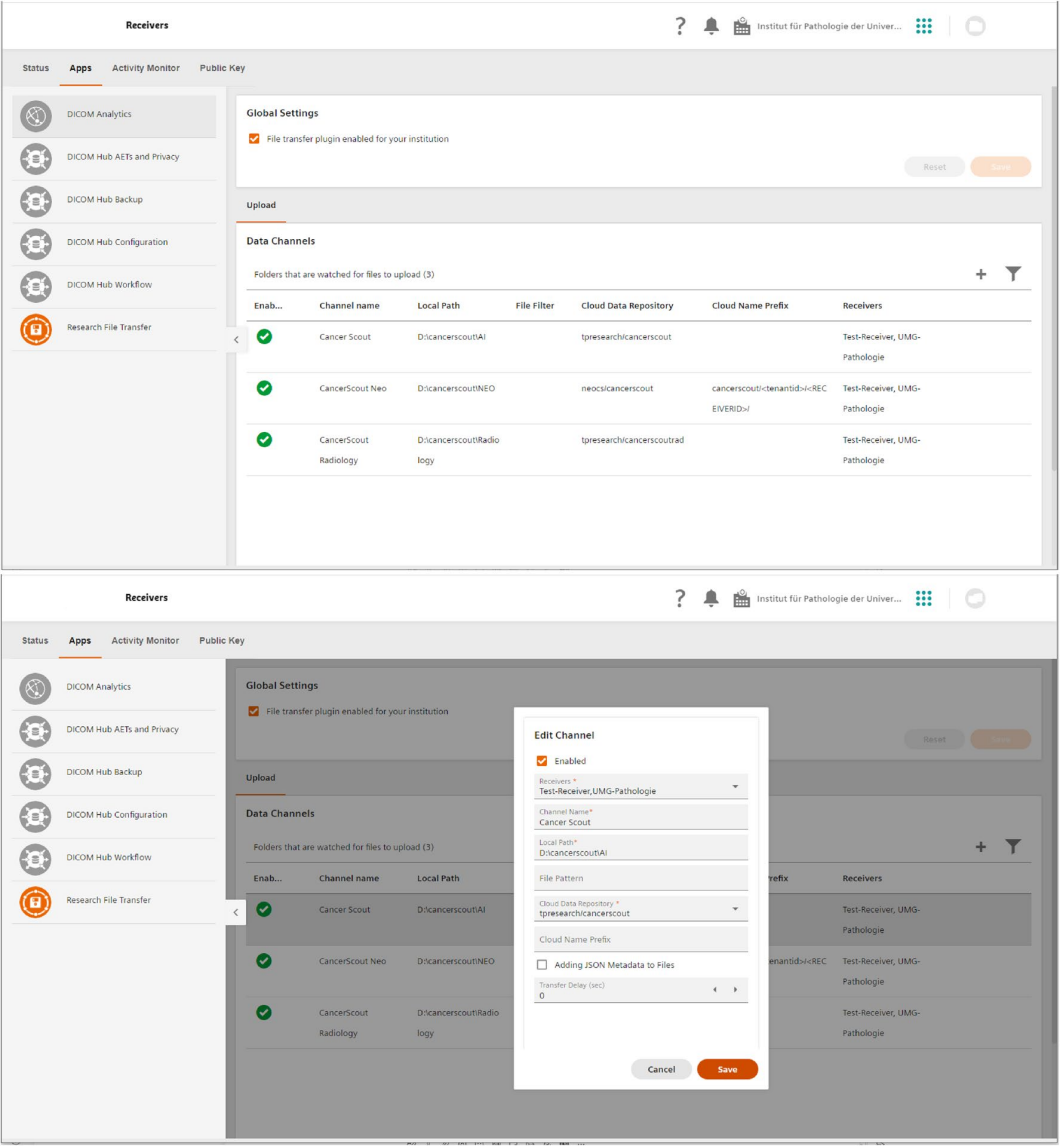
Screenshot of the cloud-based configuration for one edge-device.

In addition to the mentioned data privacy issues (see Section “Data contract”) data transfer from university medical centers (hospital) networks into cloud environments (e.g., OneDrive, GoogleDrive, Dropbox,) is often restricted by security rules such as blocked ports or firewall settings. Facilitating the firewall configurations, an edge-device was established, tunneling messages and data from and to the cloud through one connection which is secured by encryption and certificates (SSL/TLS with RSA encryption using keys with at least4096 Bit). The edge-device is setup on a virtual machine within the MeDIC as part of the research network segment, while being configured, operated, and monitored from the cloud (see Fig. 3). This enables technical IT personal to establish data channels for medical end users without on-side involvement. Focusing on the user experience for medical users, a similar approach to Microsoft OneDrive was followed, by creating local folders for each upload and download channel, which are connected to a secured cloud storage container.

For the cloud platform storage of the commercial partner, we use a similar approach to Zaharia et al.^32^ by combining Microsoft Azure Data Lake with concepts from data warehouses and direct file access for cloud data analytic platform tools (e.g., cloud hosted Jupyter Notebooks (https://jupyter.org/  [Online 2022/09/09]). While supporting ACID transactions (see Section “Cloud infrastructure supporting research”), data versioning, lineage, and metadata for each file, it also covers the requirements for handling personal data. Following the three-layer approach of data warehouses, files are uploaded to an ingestion zone which scans and associates them with a data contract before they are moved into a bronze data lake storage (layer 1: staging). From this layer the data is extracted, transformed/curated, and loaded/published by data engineers on a silver zone (layer 2). Due to the data contract reference saved in the meta data, the data privacy constraints are always known for each file regardless in which zone it is located. As big amounts of data are being processed, mounting data zones within data analytic platform tools avoids copying large files from one destination to another. Furthermore, cloud-hosted machine learning tools such as MLFlow (https://mlflow.org/ [Online 2022/08/01].) were employed, with direct file access to enable the management of the complete machine learning lifecycle.

### Technical evaluation

To show that the orchestration of the MeDIC’s clinical infrastructure together with the cloud infrastructure of the commercial partner is technically feasible, an evaluation of the approach was conducted.

Overall, data from 2000 cancer patients (treated between 2000 and 2020) were transferred from the UMG to two commercial partners. Differences were expected between small and therefore fast transferrable clinical files and large files, requiring longer transfer times. For many small files the number of parallel transfers are important, whereas large files benefit from few parallel data transfers with high bandwidth each. To evaluate both cases, we transferred whole pathology slide images and multi-omics data. The data transfer is based on Microsoft Azure platform and correspondent C# libraries - thus no problems in terms of scalability were encountered. Nevertheless, sometimes connection issues were observed and when comparing the MD5 hashes between source files and destination files large files were corrupted. The issue could be traced to regular Windows Updates, reboots of the virtual machine, and/or local IT scripts changing firewall settings. Future system designs will provide an automatic validation of source and destination file.

Within the project Cancer Scout, a research collaboration platform was used for an end-to-end machine learning lifecycle, working on a large dataset, which cannot be handled on standard local hardware. In the first step, data scientists curated raw data (bronze data lake zone) to a clean data set (silver zone) by eliminating duplicates and converting whole slide imaging (WSI) iSyntax format to Tagged Image File Format (TIFF) using cloud hosted Jupyter Notebooks. Furthermore, pathologists annotated the WSIs with cancer subtypes using the cloud-hosted EXACT tool^36^, working directly with data on the cloud data storage. The trained machine learning model for WSI classification is described in Teichmann et al.^30^. Currently, model serving is done with a cloud-based API, however, it needs to be integrated in a medical decision support tool.

## Discussion

GDPR has been introduced with the goal to protect personal data of Europeans when processed in all sectors of the economy. Notably, it fails to provide a clear instruction for processing personal data for secondary research purposes, like under which circumstances key-coded data could be considered anonymous^37,38^. Nevertheless, data collection and processing are of paramount importance for further innovation and development of the promising fields of big data and artificial intelligence in drug discovery, clinical trials, personalized medicine and medical research^39^. While secure data collection enables the collaboration between multiple public and commercial organizations to scientifically explore multi-omics data, it also facilitates medical research by using AI technologies to analyze and identify patterns in large and complex data sets faster and more precisely. From a technical point of view, the task of collecting and transferring medical data from hospitals to a collaborative cloud data platform, while ensuring privacy and security, is not trivial. To address this issue, a three layer-approach was validated, consisting of clinical data storages, a MeDIC, and a cloud platform. Firstly, the data from the clinical systems are minimized during the ETL process to the MeDIC. Secondly, each data is linked to a “data contract” when transferred from the MeDIC to the cloud, specifying data governance and defining the rights and permissions to use the data. Currently, only the trusted third party of the MeDIC can link PSN to IDAT, thus no data record linkage between different locations is possible. As linking different kinds of data from different institutions increases the risk to identify a patient (e.g., head CT, genome sequencing), this topic needs further research.

We successfully established a data platform which enables the collaboration between a public and commercial organization by enabling data transfer from various clinical systems via a MeDIC into a cloud for supporting multi-site research while ensuring compliant data governance. In a first step the approach was validated by the collaboration between one clinical institution and an industrial partner and is therefore specific to the UMG and MeDIC, as the TTP is located at the MeDIC. Based on a dataset containing 2085 diagnostic slides from 840 colon cancer patients a new AI algorithm for classification of WSI in digital pathology^30^ was proposed. Considering the literature review this implementation is, to the authors knowledge, the first work that implements this concept. To contribute the results gained from this research project the following measures were taken into account to ensure that this research meets the requirements of the FAIR principles as well. The research was made findable (F) by submitting it to a respected journal, providing a DOI (F1, F3) and further described keywords (F2). The data is findable and can be requested from the authors (F4). The data of the submission can be retrieved by the given DOI (A1), the access protocol is open, free (A1.1) and the manuscript, ones published, is accessible from different online libraries (A2). A formal language for knowledge representation was used (I1) and improved the manuscript to include vocabulary that follow the FAIR principles (I2). Furthermore, the data was described with as much information and relevant attributes (R1).

## Data Availability

The data that support the findings of this study are available from the university medical center Göttingen, but restrictions apply to the availability of these data, which were used under license for the current study, and so are not publicly available. Data are however available from the authors upon reasonable request and with permission of University Medical Center Göttingen. Please contact the corresponding author CB for all data requests.
